# Parity Deformed Jaynes-Cummings Model: “Robust Maximally Entangled States”

**DOI:** 10.1038/srep38069

**Published:** 2016-12-05

**Authors:** A. Dehghani, B. Mojaveri, S. Shirin, S. Amiri Faseghandis

**Affiliations:** 1 Department of Physics, Payame Noor University, P.O. Box 19395-3697, Tehran, I.R. of Iran; 2 Department of Physics, Azarbaijan Shahid Madani University, P.O. Box 51745-406, Tabriz, Iran

## Abstract

The parity-deformations of the quantum harmonic oscillator are used to describe the generalized Jaynes-Cummings model based on the λ-analog of the Heisenberg algebra. The behavior is interestingly that of a coupled system comprising a two-level atom and a cavity field assisted by a continuous external classical field. The dynamical characters of the system is explored under the influence of the external field. In particular, we analytically study the generation of robust and maximally entangled states formed by a two-level atom trapped in a lossy cavity interacting with an external centrifugal field. We investigate the influence of deformation and detuning parameters on the degree of the quantum entanglement and the atomic population inversion. Under the condition of a linear interaction controlled by an external field, the maximally entangled states may emerge periodically along with time evolution. In the dissipation regime, the entanglement of the parity deformed JCM are preserved more with the increase of the deformation parameter, i.e. the stronger external field induces better degree of entanglement.

The Jaynes-Cummings model (JCM) which is used extensively in quantum optics describes the interaction of a single quantized radiation field with a two-level atom. The solvability and applications of this model has long been discussed^1^,^2^. This simple model describes various quantum mechanical phenomena, for example, Rabi oscillations^2^,^3^, collapse and revivals of the atomic population inversion^4^ and entanglement between atom and field^5^. Specifically, it plays an important role in recent quantum information processing^6^,^7^,^8^,^9^,^10^,^11^. Furthermore, JCM is one of the several possible schemes for producing the nonclassical states^12^,^13^,^14^,^15^. The dynamics predicted by JCM have been proven in experiments with Rydberg atom in high quality cavities^16^. Since JCM is an ideal model in quantum optics, its various extensions such as intensity dependent coupling, two photons or multi-photon transitions, two- or three- cavity modes for three-level atoms and the Tavis-Cummings model have been proposed^17^,^18^. In 1984, Sukumer and Buck studied the above mentioned models by using algebraic operator methods^19^. On the other hand, the supergroup theoretical approach to JCM leads to the exact solvability of this model and the representation theory of super-algebras^20^. More recently, a lot of researchers have found that the ordinary creation and annihilation operators in JCM may be replaced by the q-deformed partners, namely, the *q*-deformed JCM^21^. Later on, JCM has been adopted with a Kerr nonlinearity within the framework of *f*-oscillator formalism^22^. Furthermore, the investigations of a class of shape-invariant bound state problem, which represents a two-level system, leads to the generalized JCM^23^.

In addition to the above -cited generalizations, in recent years, a lot of interest has been given to to the extension of the boson oscillator algebra. One of the most interesting cases which is not related to the *q* or *f*-calculus is *R*-deformed Heisenberg algebra (RDHA). Such generalized schemes naturally lead to the concept of para-fields and para-statistics^18^,^19^,^20^,^21^,^22^,^23^,^24^,^25^,^26^,^27^,^28^,^29^,^30^,^31^,^32^,^33^,^34^,^35^,^36^. The same RDHA was also used for solving the quantum mechanical Calogero model or pseudo harmonic oscillator (PHO)^37^,^38^,^39^,^40^,^41^. Recently, this algebra has been employed for bosonization of super-symmetric quantum mechanics^41^,^42^,^43^ and for describing anyons in (2 + 1)^42^,^44^ and (*l* + 1) dimensions^45^,^46^. All the applications as well as the para-bosonic constructions^32^,^33^ have used infinite-dimensional unitary representations of RDHA. According to Wigner’s new quantization method, the RDHA is raised as a unital algebra by the generators 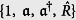, which satisfy the (anti-) commutation relations


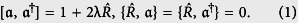


Here, 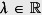 is called Wigner’s deformation parameter and 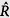 is a parity operator with the following properties


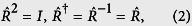


This acts in the Hilbert space of eigenfunctions as follows


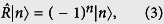


which means that 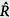 commutes with number operator 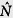 that includes the eigenvector 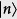, such that 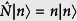. The number operator 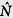 is in general different from the product 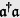, and is postulated to satisfy the following relations:


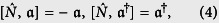



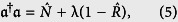


which provide us with the following irreducible representation of the RDHA


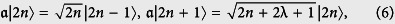



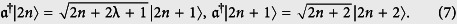


The explicit differential forms of the generators 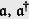 in terms of the well known annihilation and creation operators *a*, *a*^†^ are obtained^37^ as follows:


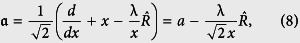



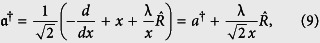


which provide us with the coordinate representation for the position 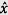 and its *λ*−deformed canonical pair 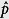 as


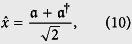



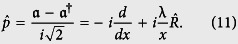


It is evident that the above representation is really different from the *f*- deformed realization of the Heisenberg algebra^47^. Therefore, the introduced RDHA in (1) can be considered as a new deformation of the simple harmonic oscillator with significant features in quantum optics^48^,^49^.

Due to the physical significance of the deformed JCM in quantum optics on the one hand, and the central role of the parity operator in the theory of deformation on the other hand, we then generalize the well-known JCM to a parity deformed-Hermitian case in terms of parity deformed boson operators which are not related to the *q* or *f*-deformation. It should be noted that the parity deformed JCM introduced here could be compared, with a major difference, to what has been already discussed in other studies as the *q*-, *f*-deformed JCM and so on. It is one of the most interesting cases of the parity operator 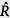 appeared in the context of quantization schemes generalizing bosonic commutation relations. These Hermitian operators arise from a special deformation of canonical bosonic commutation relations, allowing us a mathematically rigorous treatment of our deformed interaction Hamiltonian, extraction of the energy spectrum, and the corresponding eigen-vectors. Preparing the initial field in the *λ*− deformed cat states, we investigate the collapse and revival phenomena in the Rabi oscillations of the atomic inversion.

Considering the experimental realization, interaction with the environment is an unavoidable feature of real quantum systems. Dissipation phenomenon of energy into the environment is the main feature of such real systems which leads to the loss of entanglement generated in those systems. Some scholars have studied this subject and found interesting phenomena such as entanglement revival^50^, entanglement sudden death^51^,^52^ and sudden change for quantum discord^53^. The description of dissipative systems Hamiltonian is a polemic topic in the literature^54^,^55^. The dissipative systems can be studied in a phenomenological way in which a closed system formed by the cavity including its environment is considered. This larger system is therefore closed, and it is possible to apply quantum mechanics to it in a usual way^56^. The mentioned environment can be regarded as a discrete set of degrees of freedom, or a set of harmonic oscillators^57^. In addition, the surrounding environment can be modeled as a set of continuum harmonic oscillators^58^. The Hamiltonian describing this model of dissipation is called the Gardiner-Collett Hamiltonian^59^. Also, based on the dissipation of the atomic upper level, a model describing dissipative atom-field system is introduced^60^. This model described by a non-Hermitian Hamiltonian is called damping JCM. The authors have studied decaying behaviour of the entanglement between atom and field. Also, one of the interesting topics in dissipative systems is to find a way to fight against the decay of the entanglement. Many schemes have been proposed in order to preserve entanglement in such systems. For instance, it has been shown that, the addition of a laser field leads to high stationary entanglement^57^. Another approach to overcome this problem, relies on active feedback^61^,^62^,^63^. In addition to these, the quantum Zeno effect is a promising way to avoid the decaying behaviour of the entanglement in dissipative systems^64^,^65^,^66^,^67^,^68^. In the dissipation regime, we use the damping deformed JCM to describe a two-level atom interaction with the dissipative cavity and investigate individually and simultaneously the effects of dissipation and detuning parameters on the entanglement between the two-level atom and the deformed field. It has been shown that by detuning modulation, and setting the deformation parameter, coupling constant and average photon number of the field, the degree of entanglement of the atom-field states, and the atomic population inversion may be controlled.

## Parity Deformed JCM and Interpretation

The system we introduce here is a parity extension of the standard Jaynes-Cummings model in the rotating-wave approximation, as follows


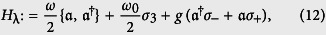


along with substitution Eqs (8) and (9) in Eq. (12), we can recast the Hamiltonian *H*_λ_ into


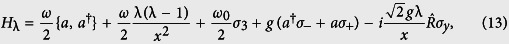



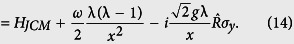


It recalls a system of a two-level atom coupled simultaneously to a single-mode of the quantized electromagnetic field and a centrifugally external classical field. It reduces to an ordinary JCM, while *λ* → 0. Here, *σ*_±_ and *σ*_3_ are usual rising (lowering) and inversion operators for the atomic states, |±〉, satisfying [*σ* + , *σ*_−_] = *σ*_3_ and [*σ*_3_, *σ* ±] = ±2*σ*_±_, *a*^†^ and *a* are the creation and annihilation operators for the cavity mode [*a*, *a*^†^] = 1, *g* is the coupling constant between the atom and the cavity field mode, *ω* and *ω*_0_ are the cavity as well as the external mode frequency and the atomic transition frequency, respectively, *λ* represents the strength of the external field, 
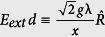
 is the coupling constant between the atom and the external classical field where *d* recalls the atomic dipole matrix element for the transition and *E*_*ext*_ refers to the amplitude of the external field. It is noteworthy that the Hamiltonian *H*_*λ*_ is super-symmetric when *ω* = *ω*_0_ (exact resonance) and *g* = 0 (absence of coupling). This exactly solvable model includes the inversely quadratic potential, 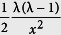, first proposed by Post in 1956 when he studied the one-dimensional many identical particles problem in the case of the pair-force interaction between the particles^69^. In addition to the two typical model potentials such as the Morse potential and Poschl- Teller potential, this an-harmonic oscillator potential can be considered as a good candidate to describe the molecular vibrations too^70^,^71^,^72^. Since 1956, this quantum system was studied by other researchers, extensively. For example, Landau and Lifshitz studied its exact solutions in three dimensions^73^. Recently, Sage has studied the vibrations and rotations of the pseudo-gaussian oscillator in order to describe the diatomic molecule^74^, in which he briefly reinvestigated some properties of the PHO in order to study the pseudo-gaussian oscillator. Advantages of the pseudo-harmonic potential have been considered for improvements in the conventional presentation of molecular vibrations^75^. Hurley found that this kind of PHO interaction between the particles can be exactly solved by the separation of variables while studying the three-body problem in one dimension^76^. A few years later, Calogero studied the one-dimensional three- and N-body problems interacting pairwise via harmonic and inverse square (centrifugal) potential^77^,^78^. On the other hand, this potential was generalized by Camiz and Dodonov *et al*. to the non-stationary (varying frequency) PHO potential^79^,^80^. In addition, such a physical problem was also studied in arbitrary dimension. Also, Dong *et al*. have studied its dynamical group in two dimensions^81^.

This parity deformed JCM model possesses an exact solution because of the existence of an integral of motion, 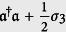, which commutes with the Hamiltonian *H*_λ_ and allows us to decompose all the representation space of the atom-field system as the tensor product of the Hilbert space associated with the field, 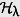, times the Hilbert space associated with the spin, 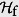,


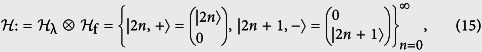


or


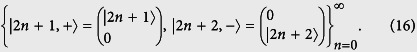


Here, 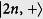 is the bare state in which the atom is in the excited state 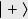 and the field has 2*n* photons, and a similar description holds for the bare state 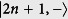, where 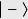 is the atom ground state. Using the Fock space 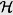 given in (15), we can find the following matrix representation of the *λ*−deformed JC Hamiltonian *H*_λ_:


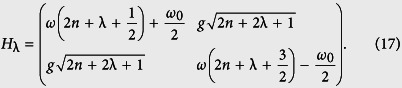


It is easy to see that the corresponding dressed eigen-states of *H*_*λ*_ are


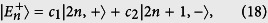



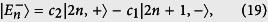


where the coefficients *c*_1(2)_ are given by:


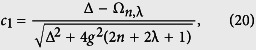



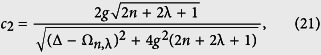


in which Δ(= *ω* − *ω*_0_) and 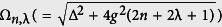 are defined as detuning parameter and a generalized Rabi frequency, respectively. The energy eigenvalues corresponding to the eigen-states in Eqs (18) and (19) are


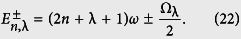


**Figure 1:**
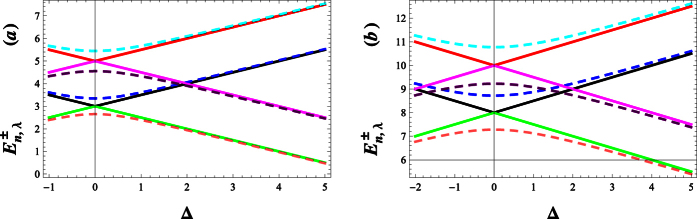
Dependence of eigenvalues 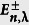 on detuning Δ. The continuous curve corresponds to *g* = 0.01. The dashed curves (**a**) and (**b**) correspond to (*g* = 0, λ = 0) and (*g* = 0, λ = 50), respectively. The dashed curves with positive and negative slopes correspond respectively to 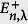 and 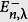. Lower part of the figure is for *n* = 1 and upper part for *n* = 2.

The energy difference between the levels 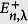 and 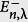 is Ω_*n*,λ_. The minimum of the separation occurs when Δ equals to zero and the corresponding difference is 
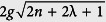
. In Fig. 1(a) and (b), respectively, we have plotted the energy eigenvalues 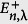 and 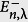 as functions of Δ for given values of λ = 0, 50. The dotted lines represent the eigenvalues when *g* = 0, i.e. 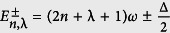. In this case, the eigenvalues cross each other as they increase from negative to positive values. The continuous lines represent the energy eigenvalues for *g* = 0.01. The diverging eigenvalue separation beyond the minimum separation indicates level repulsion in the eigenvalues of the dressed states. As Fig. 1(a) and (b) show, the repulsion between energy levels increases as the deformation parameter λ gets bigger. The latter, also, leads to shift the energy levels to the positive side.

## Evolution of Atom-Field State

In order to study the influence of the deformation on the dynamics of the parity-deformed JCM, firstly we decompose the Hamiltonian (12) as follows:


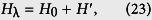


where


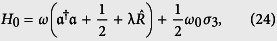



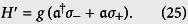


In the interaction picture generated by *H*_0_, the Hamiltonian of the system can be written as


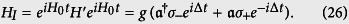


We now proceed to solve the equation of motion of this system in an interaction picture, that is


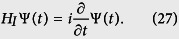


At any time *t*, the wave function Ψ(*t*) is expanded in terms of the states 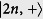 and 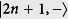 as follows


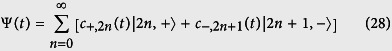


Clearly, Ψ(*t*) is determined completely once the coefficients *c*_+,2*n*_(*t*) and *c*_−,2*n*+1_(*t*) are known. Inserting (28) into (27), we obtain the following general solution for the probability amplitudes, *c*_+,2*n*_(*t*) and *c*_−,2*n*+1_(*t*), as:


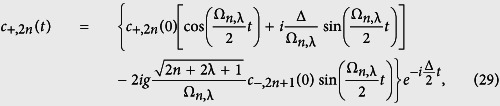



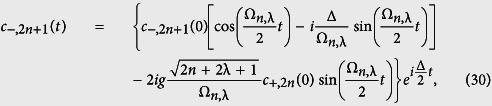


where the constants *c*_−,2*n*+1_(0) and *c*_+,2*n*_(0) are determined from the initial conditions of the system, which is supposed initially in the upper level, i.e. *c*_+,2*n*_(0) = *c*_2*n*_(0) and *c*_−,2*n*+1_(0) = 0. Here, the initial condition for the field is described by *c*_2*n*_(0). For this case in particular, we have


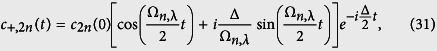



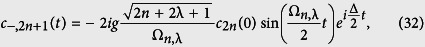


This set of equations gives us the solution for the problem. In order to calculate some physical quantities of interest, we need only to specify the initial photon number distribution of the field |*c*_2*n*_(0)|^2^.

The field we are considering in this work is being treated as an λ-deformed oscillator which could be described in different ways. We focus on the situations in which the field as an eigen-state of the λ-deformed annihilation operator is introduced^48^, i.e. 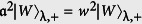, and its number state expansion is


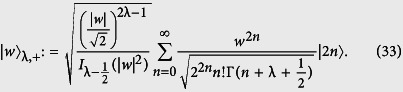


Therefore, in this case, we have


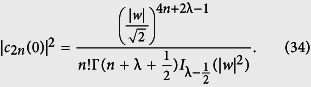


It needs to be noted that, as a second option, one may choose the initial state of the field as the Wigner negative binomial states already studied^49^. They are mathematically equivalent to those nonlinear ones and may be expected to bring new quantum features.

## Atomic Dynamics and Dissipative Limit

In this section, we are interested in studying the temporal evolution of atomic inversion, which, in turn, is specified by the expectation value of the inversion operator as:


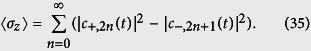


Substituting Eqs (31), (32) and (34) into (35), we obtain, for an atom prepared initially in the excited state,


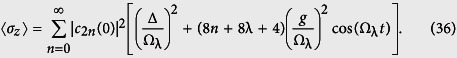


**Figure 2:**
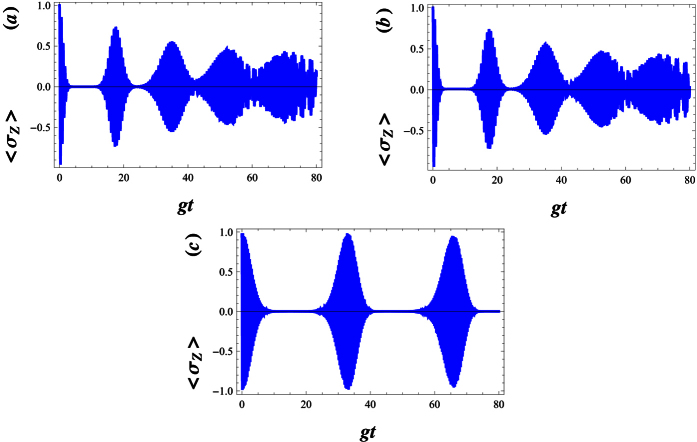
Temporal evolution of the atomic inversion 〈σz〉 for the field initially prepared in WCS, 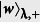, with |w|^2^ = 30 and g = 0.01. The parameters are (**a**) λ = 0, Δ = 0, (**b**) λ = 0, Δ = 0.01 and (**c**) λ = 50, Δ = 0.01.

**Figure 3:**
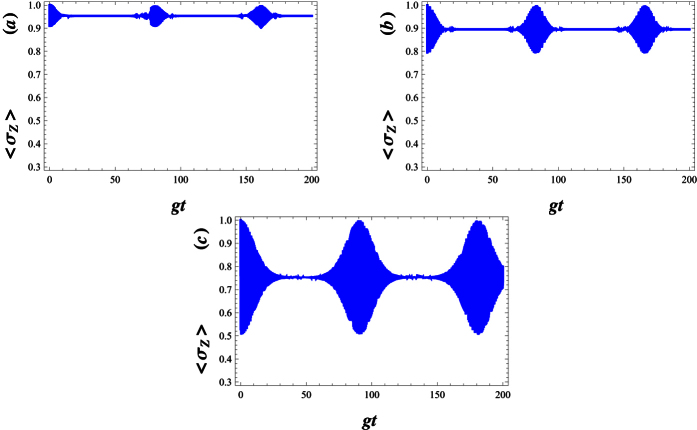
Temporal evolution of the atomic inversion with |*w*|^2^ = 30, *g* = 0.01 and Δ = 0.5. The parameters equal to (**a**) λ = 0, (**b**) λ = 30 and (**c**) λ = 100.

The numerical results of the atomic inversion when the field is in a standard cat state (i.e. when λ = 0), have been shown against the scaled time *gt* in Fig. 2(a) and (b), respectively. The temporal evolution of the atomic inversion 〈*σ*_*z*_〉 reveals significant discrepancies of the well-known phenomenon of collapses and revivals^82^. Note that the collapse, i.e. when the envelope of the oscillations collapses to zero, is due to the destructive interference among the probability amplitudes at different Rabi frequencies, Ω_*n*,λ_, for different photon number eigen-states. At the revival times, on the other hand, constructive interference occurs. This phenomenon also takes place when the initial field state is a WCS. In Fig. 2(c), we have pictured the function 〈*σ*_*z*_〉 for the value λ = 50. In this case, 〈*σ*_*z*_〉 exhibits quasi periodic behaviour very similar to the atomic inversion of a two-photon JCM^83^,^84^,^85^, with an effective Hamiltonian defined as 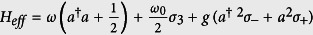. However, in this case, note how the structure of the oscillations is much more complex than the standard Rabi oscillations. As the detuning factor Δ increases, these structures are disappeared (see Fig. 3), i.e. the inhibition of the radiation decay is more transparent. It is clear that the inhibited decay even occurs in the case *λ* = 0. This behaviour is due to the influence of the parity deformation via the generalized Rabi frequency Ω_*n*,λ_. Figure 3(a–c) indicate that, with increasing λ the inhibition decay of the excited state will be balanced.

**Figure 4:**
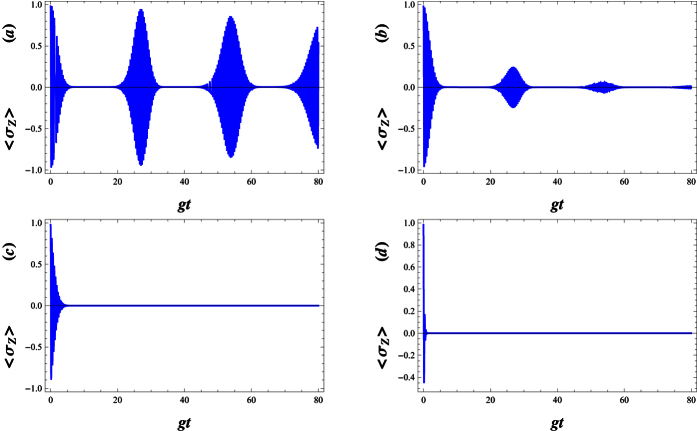
Temporal evolution of the atomic inversion with |*w*|^2^ = 30, *g* = 0.01, Δ = 0.5 and λ = 30. The parameters equal to (**a**) *γ* = 0, (**b**) *γ* = 0.001, (**c**) *γ* = 0.01 and *γ* = 0.1.

**Figure 5:**
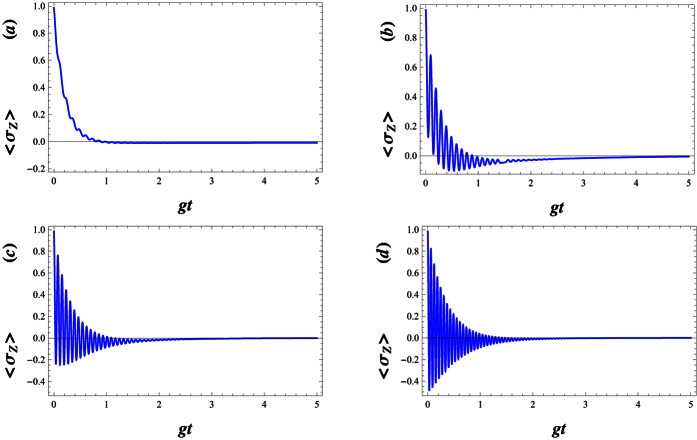
Temporal evolution of the atomic inversion with |*w*|^2^ = 30, *g* = 0.01, Δ = 0.5 and *γ* = 0.05. The parameters equal to (**a**) λ = 0, (**b**) λ = 200, (**c**) λ = 500 and λ = 1000.

The finite lifetime of the atomic levels can be described by adding phenomenological decay terms, 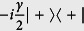, to the Hamiltonian (9), where 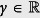 is a decay constant^60^. For an atom initially stated in the upper level, |+〉, the population inversion dynamics are analyzed for a deformed JCM was surrounded by a dissipative environment where the dissipation of the upper-level is considered (see Fig. 4). Here, we plot the dynamics of the population inversion in the presence of the decay term against the scaled time *gt*. In Fig. 4, we compare the effects of increasing *γ* on the Rabi oscillations, where the latter is destroyed. However, as is illustrated in Fig. 5, the patterns of the revivals are restored while λ is enhanced.

## Fidelity

**Figure 6:**
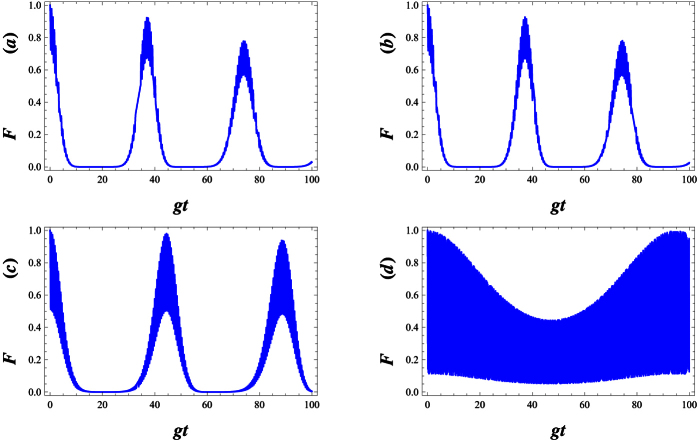
Fidelity as a function of the scaled time of *gt* in the small coupling regime with *g* = 0.01 for different λ( = −0.25, 0, 10, 100), other parameters are |*w*|^2^ = 9 and Δ = 0.1.

We now calculate the fidelity


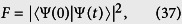


which measures the “closeness” of the two quantum states 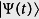 and 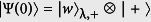, which indicate that *F* is unity when these two quantum states are identical. Figure 6 indicates that with increasing λ the fidelity decreases but remains close to its initial value (see Fig. 5(a)–(d)). To obtain fidelity around 1, one needs to enhance the deformation parameter λ to 100 and *gt* = 95. In this case, 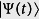 becomes minimum uncertainty state which minimize the uncertainty relation, Eq. (28) in ref. 48.

## Generation of Maximally Entangled States

Entanglement is a striking feature of the quantum mechanics can be compared with the classical ones^86^. This phenomenon as a nonlocal correlation between two (or more) quantum systems plays a fundamental role in the quantum information science such as quantum computation and communication^87^,^88^,^89^, teleportation^90^, dense coding^91^,^92^, cryptography^93^ and etc. Generally, characterization of amount of entanglement is achieved through the well justified and mathematically tractable measures^94^. For bipartite states, a number of acceptable entanglement measures such as the entanglement of formation and distillation^95^, concurrence, negativity^96^, Von Neumann entropy and relative entropy^97^ have been proposed. In this section, in order to obtain the degree of entanglement between atom and field, we choose the Von Neumann entropy. The Von Neumann entropy is a very useful operational measure of the disorder of a system and of the purity of a quantum state. For given density operator *ρ* the Von Neumann entropy is given by


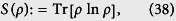


where “Tr” often abbreviated to the trace and *S*(*ρ*) ranges from 0 for a separable state to 1 for a maximally entangled one. As the deformed JCM is a bipartite system, a Schmidt decomposition is assured. Based on the solution of the time-dependent Schrodinger equation (23), and according to the Schmidt decomposition, for any instant in time *t*, we can always find the reduced density operator of the atom in the bare basis specified by |±〉 and obtain


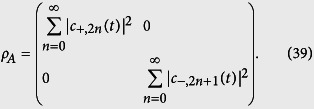


Clearly, eigenvalues of the density operator for the atom, *g*_±_, can be expressed in terms of the coefficients *c*_+,2*n*_(*t*) and *c*_−,2*n*+1_(*t*) i.e. 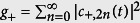 and 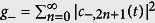. Then, it is easy to obtain an expression for the Von Neumann entropy. For atomic subsystem, this is:


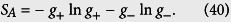


**Figure 7:**
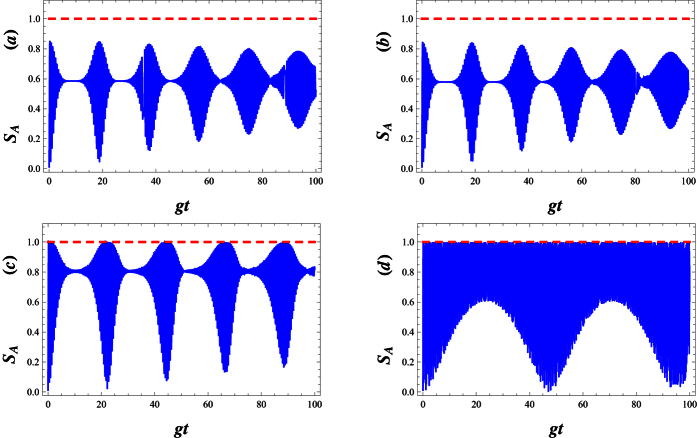
Plots of entropy *S*_*A*_ versus *gt* with *g* = 0.01, |*w*|^2^ = 9 and Δ = 0.1 for various deformation parameters respectively (**a**) λ = −0.25, (**b**) λ = 0, (**c**) λ = 10 and (**d**) λ = 50.

**Figure 8:**
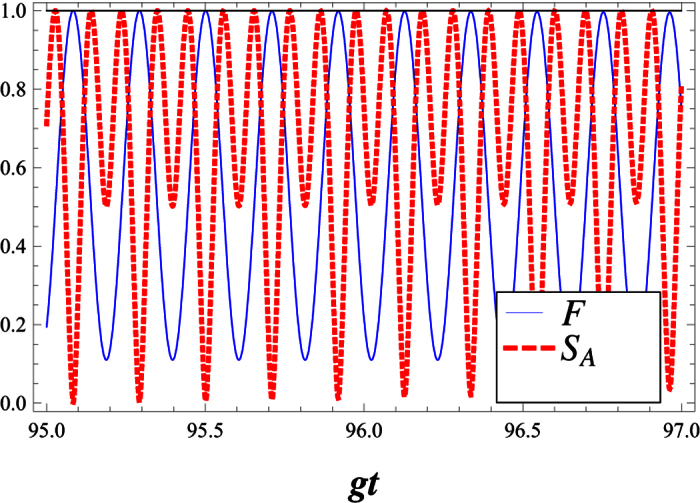
The Von Neumann entropy (dash) and the fidelity (solid) are plotted together. In each case a deformation parameter with λ = 100, *g* = 0.01, |*w*|^2^ = 9, Δ = 0.1 is used.

In Fig. 7, for an atom initially in an excited state and the field initially in WCS, we plot the Von Neumann entropy *S*_*A*_, all against the scaled time *gt*. We can also see that the entropy makes quasi-period oscillation. This means that the deformed field can help to realize and stabilize the degree of entanglement between the atom and the field at a high level. Sometimes, as the deformation parameter λ is enhanced, the atom- field system becomes maximally entangled (Fig. 7(c) and (d)). In Fig. 8, we compare the Von Neumann entropy and fidelity of the quantum state, Ψ(*t*), associated with the λ−deformed JCM investigated here. Bear in mind that entanglement between the atom and field is maximized i.e. the quantum state Ψ(*t*) becomes maximally entangled, where fidelity of the quantum state passes the smallest value.

## Dissipative Regime and Robust Entangled states

**Figure 9:**
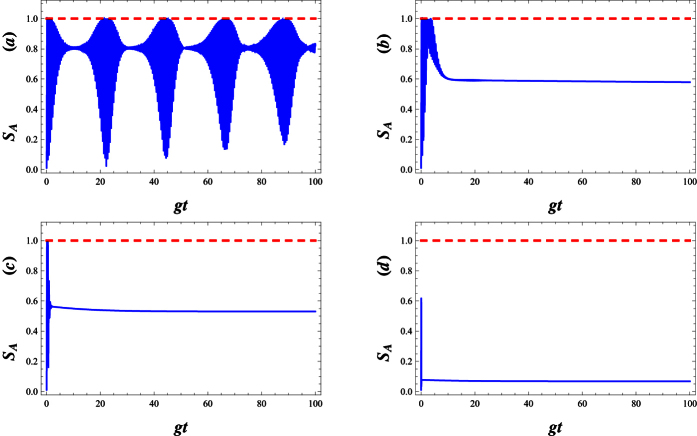
The time evolution of atomic entropy *S*_*A*_ in the dissipative regime for *g* = 0.01, |*w*|^2^ = 9, Δ = 0.1 and λ = 2, (**a**) *γ* = 0, (**b**) *γ* = 0.01, (**c**) *γ* = 0.1 and (**d**) *γ* = 1.

Now, we consider the dynamics of entanglement between atom and field in dissipative regime. For this purpose, the time evolution of the Von Neumann entropy for different decay coefficients near the resonant case has been plotted in Fig. 9. In an ideal case which no decay rate is considered, as is shown in Fig. 9(a), *S*_*A*_ suddenly increases from 0 to its maximum value and then the collapse and revival patterns around the 0.8 (a amount near its maximum value) are presented. In the actual system, the atom is steady when it is in the ground state. However, when the atom is in the excited state, some factors such as the spontaneous emission, the collision between atoms and so on, will lead to the decay of the upper-level. In this case, *S*_*A*_ attains a stable behaviour after some fluctuations at the beginning of the interaction (see Fig. 9(b–d)). As is seen, the increment of the decay coefficients not only disappears the rapidly oscillations of *S*_*A*_ in the initial times, but also leads to a reduction of the amount of entanglement.

**Figure 10:**
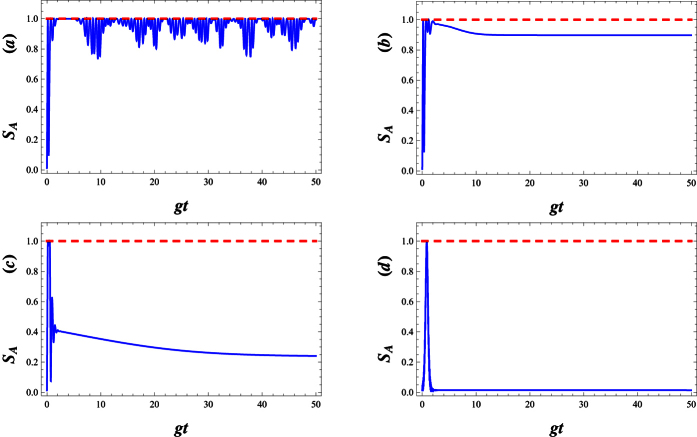
The time evolution of atomic entropy *S*_*A*_ in the dissipative regime with *g* = 0.01, |*w*|^2^ = 9, λ = 2 and *γ* = 0.1, (**a**) Δ = 0, (**b**) Δ = 0.01, (**c**) Δ = 0.1 and (**d**) Δ = 1.

In Fig. 10, to investigate the influence of the field frequency modulation on the atom-field entanglement, we set different modulation frequencies. When we compare Fig. 10 with Fig. 9, we find that the entanglement can last a longer time as Δ decreases. The entanglement decays to zero in the larger detuning parameter while the entanglement still exists in the resonant case. However, the maximum of the entropy decreases with an increase in Δ (see Fig. 10(b)). The small detuning, Δ < 1, reduces the maximum value of entanglement in the short time region, while it prolongs the entanglement time. Comparison of Fig. 10(b) with Fig. 10(d), reveals that larger modulation is unfavorable to the maintenance of the entanglement for a long time.

**Figure 11:**
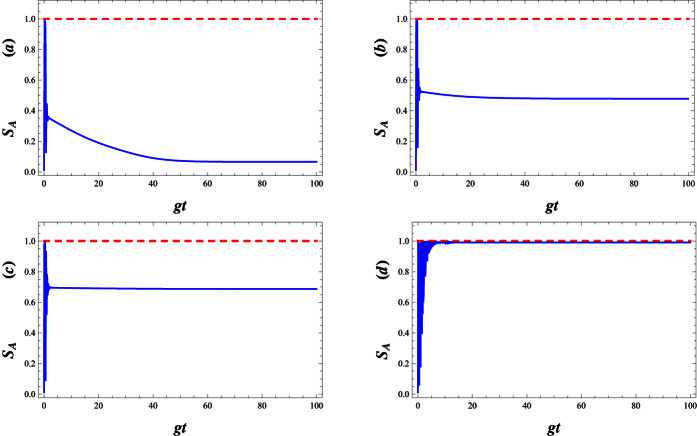
The time evolution of atomic entropy *S*_*A*_ in the dissipative regime with *g* = 0.01, |*w*|^2^ = 9, *γ* = 0.1 and Δ = 0.1, (**a**) λ = 0, (**b**) λ = 10, (**c**) λ = 20 and (**d**) λ = 80.

Fig. 11 refers to the effect of the parity deformation on time evolution of the entanglement. As is shown, in the absence of λ, the atomic entropy after suddenly increasing to its maximum value at the beginning of the interaction, decay to a minimum asymptotic (stable) value in enough large times (see Fig. 11(a)). As is seen, the presence of λ not only decreases the decay time but also causes the enhancement of the asymptotic (stable) values of atomic entropy (see Fig. 11(c–d)) in which for large values of λ, the stable values of entanglement reaches to 1. On the other words, by increasing *λ* the generated maximally entangled states are preserved, in the dissipation regime at the beginning of the interaction.

## Non-classical Properties: Sub-Poisonian Statistics and Squeezing Effect

We now examine the time evolution of the nonclassical properties of the constructed states Ψ(*t*). To achieve this purpose, we investigate the sub-Poissonian statistics and their quadrature squeezing. It should be mentioned that squeezing or sub-Poissonian statistics are sufficient requirements for a state to belong non-classical ones. The anti-bunching effect as well as the sub-Poissonian statistics of the states Ψ(*t*) is investigated by evaluating Mandel’s *Q*^λ^ parameters, which are defined as


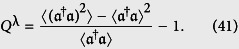


**Figure 12:**
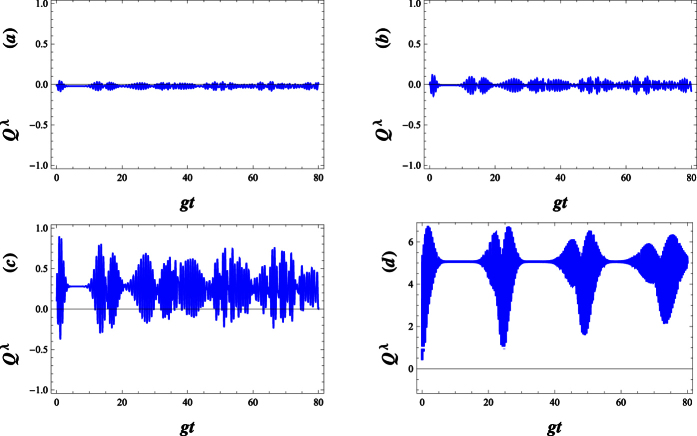
Plots of the normalized variance, Q^λ^, as a function of the normalized time gt, for the field initially prepared in an annihilation operator coherent state 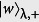 with |w|^2^ = 20, g = 0.01 and Δ = 0.01. The parameters are (**a**) *λ* = −0.25, (**b**) *λ* = 0, (**c**) *λ* = 2 and (**d**) *λ* = 10.

The inequality *Q* < 0 indicates the sub-Poissonian photon number distribution, which implies that photons are antibunched. It is well know that sub-Poissonian statistics is a signature of the quantum nature of the field. Conversely, *Q* > 0 holds for a super-Poissonian photon number distribution. Also, *Q* = 0 corresponds to the canonical coherent state. Here, the angular brackets denote averaging any field operator 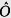 over an arbitrary normalizable state Ψ(*t*) for which the mean values are well defined, i.e.


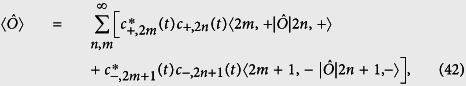


where the probability amplitudes *c*_+,2*n*_ and *c*_−,2*n*+1_(*t*) are given by equations (26) and (27), respectively. In Fig. 12, we have shown the temporal evolution of the Mandel’s *Q*^*λ*^ parameters given by equation (41), when the field is initially in the WCS. It has been demonstrated that its statistics tend to fluctuate around Poissonian conduct for small values of λ(see Fig. 12(a) and (c)). On the other hand, the statistics of the system exhibits, in general, a super-Poissonian behaviour for the same |*w*|^2^ initial value while λ > 0.

Squeezing of radiation is a purely nonclassical phenomenon without any classical analogue and has attracted considerable attention owing to its low-noise property. It has been either experimentally observed or theoretically predicted in a variety of nonlinear optical processes. Now, let us consider the squeezing properties of the field by introducing the following two Hermitian field amplitudes, 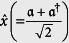 and 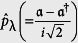. The uncertainty relation for the variances of these operators are obtained as


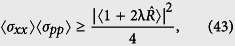


where 
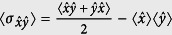
 and the angular brackets denote averaging over an arbitrary normalizable state for which the mean values are well defined, 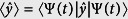. It can be said that a state is squeezed if the condition 
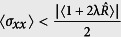
 or 
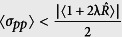
 is fulfilled^98^,^99^. In other words, a quantum state is called squeezed state if it has less uncertainty, in one parameter (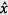 or 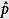), than a coherent state. Then to measure the degree of squeezing, we introduce the squeezing factors *S*_*x*(*p*)_^100^, corresponding with the state Ψ(*t*), respectively


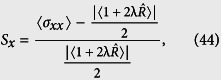



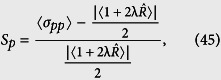


which results that the squeezing condition takes the simple form 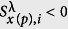. By using the mean values of the generators of the WHA,


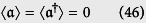



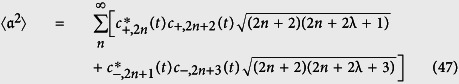



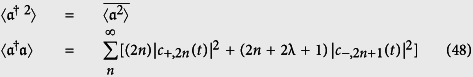



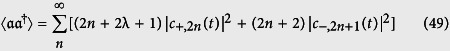



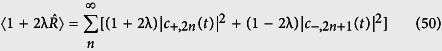


one can derive the variance and covariance of the operators 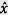 and 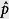. From Eqs (44) and (45), we also stress that the squeezing is very sensitive to the deformation parameter λ, which can be discussed as follows

**Figure 13:**
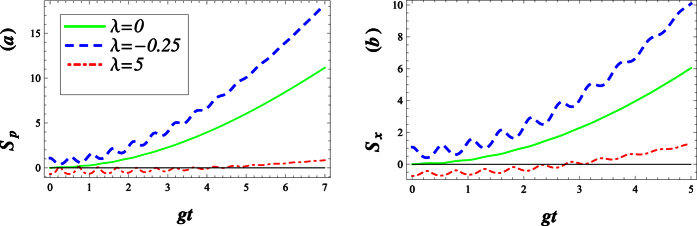
Squeezing in the *p* and *x* quadratures against *gt* for different values of *λ* with *g* = 0.01, |*w*|^2^ = 9 and Δ = 0.1 as well as for fixed values of *ϕ* = 0 and 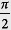 correspond with (**a**) and (**b**), respectively. The solid curve is plotted for λ = 0.

- Figure 13(a) and (b) visualize variations of the squeezing factors *S*_*p*_ and *S*_*x*_ in terms of *gt* for different values of the deformed parameter λ = −0.25, 0 and 5 when we choose the phase *ϕ* = 0 and 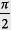, respectively. These show that the squeezing effect in the field operator *p* may be considerable for *ϕ* = 0 and small values of *gt* while λ > 0. As seen in Fig. 13(a), the squeezing factor *S*_*p*_ tends to zero which indicates that the states Ψ(*t*) become minimum uncertainty ones.

- For the case *ϕ* = 0, our calculations show that the squeezing factors *S*_*p*_ are really dependent on λ. Figure 13(a) shows that, with a rise in λ, the degree of squeezing or depth of non-classicality increases at first and then decreases when time goes on.

- Squeezing in the *p* quadrature disappears when *ϕ* reaches 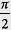, where squeezing in the *x* quadrature is raised (see Fig. 13(b)).

## Conclusions

A parity deformed Jaynes-Cummings Hamiltonian in terms of spin and λ-deformed bosonic operators, was introduced. Its eigen-states and eigenvalues were obtained explicitly. Mathematical and physical implications and applications of our results were discussed in detail. The deformed JCM introduced here may add new insights to nonclassical states of radiation in cavity QED. It, also, can be used to further investigate the interaction between an atomic system and a single mode of an electromagnetic field, including damping or amplifying processes, which are of fundamental importance, in quantum optics. By assuming that the atom is initially prepared in the excited state and the field is in the WCS, its quantum dynamical features such atomic inversion, quantum statistics and squeezing of obtained wave functions of the system were investigated. It was found that the atomic inversion exhibits Rabi oscillations including quasi-periodic behavior. Further examination of non-classical signs of the atom-field states, revealed that a significant squeezing can be achieved for positive deformation parameters. Furthermore, increasing the deformation parameter (the stronger external field) changes their statistics from the Poissonian to super-Poissonian. The λ- deformed JCM, driven JCM, can be applied to generate maximally entangled states. In other words, the small detuning and coupling regimes with a large deformation parameter may lead to a long-lasting robust maximally entangled quantum state. It was illustrated that for large values of λ, the generated maximally entangled quantum state was preserved as time goes on, despite the presence of the dissipation. It is worth mentioning that the approach presented here can be potentially compared with some others, already discussed in the literature, where other researchers tried to generate and stabilize the entanglement. In other words, we investigate how an appropriate choice of the external field allows one to control atom-field entanglement. Finally, a possible generalization to the three-level system can be discussed.

## Additional Information

**How to cite this article**: Dehghani, A. *et al*. Parity Deformed Jaynes-Cummings Model: “Robust Maximally Entangled States”. *Sci. Rep.* **6**, 38069; doi: 10.1038/srep38069 (2016).

**Publisher's note:** Springer Nature remains neutral with regard to jurisdictional claims in published maps and institutional affiliations.
